# DC-Compensated Current Transformer †

**DOI:** 10.3390/s16010114

**Published:** 2016-01-20

**Authors:** Pavel Ripka, Karel Draxler, Renata Styblíková

**Affiliations:** 1 Faculty of Electrical Engineering, Czech Technical University, Technicka 2, 166 27 Praha 6, Czech Republic; draxler@fel.cvut.cz; 2 Czech Metrology Institute, V Botanice 4, 150 72 Praha 5, Czech Republic; rstyblikova@cmi.cz

**Keywords:** current transformer, current sensor, DC tolerance

## Abstract

Instrument current transformers (CTs) measure AC currents. The DC component in the measured current can saturate the transformer and cause gross error. We use fluxgate detection and digital feedback compensation of the DC flux to suppress the overall error to 0.15%. This concept can be used not only for high-end CTs with a nanocrystalline core, but it also works for low-cost CTs with FeSi cores. The method described here allows simultaneous measurements of the DC current component.

## 1. Introduction

Instrument current transformers (CTs) can be heavily influenced by the DC component in the measured current [1,2,3]. This has become a serious problem, as DC currents are very common in the power grid. Historically, they have been created by geomagnetic storms. However, in the recent decade they have more often been created by transformerless power inverters, which have become standard in solar and wind power plants [4]. The DC current component of these inverters is usually compensated by a feedback loop controlled by the DC current sensor. Most of these sensors are Hall effect devices, which have large drift with temperature and time, leading to DC compensation failure. A significant DC component is also caused by halfway rectifiers, which are used by cheating customers to lower their electricity bill. In this case, the DC current component is 60% of I (50 Hz).

Bipolar saturation of CTs can be detected numerically [5], and primary current information can be recovered by various software techniques [6,7] and hardware techniques [8,9]. Only a small number of authors have applied similar techniques for the case of unipolar saturation [10].

In this paper, we discuss existing techniques for increasing the DC current resistance of CTs, and we present a method for suppressing DC magnetization by hardware feedback.

DC current tolerance is a well-known issue in domestic power meters which can be tampered by halfway rectification on the consumer side. DC-tolerant CTs utilize two technologies:

1. A composite transformer core consisting of a high-permeability core and high-saturation core. It was shown in [11] that these cores can fail if the power factor is significantly lower than 1. This effect can only be partly compensated by numerical correction of the phase delay [12].
2. High-saturation cores made by stress annealing of nanocrystalline tapes. Stress annealing can be performed on wound cores [13] or continuously on tape before winding [14], despite the brittleness of nanocrystalline materials. Stress annealing introduces anisotropy with the easy axis perpendicular to the tape length. This decreases the coercivity and permeability, makes the magnetization characteristics linear, and increases the saturation field. Due to their low permeability, these cores exhibit a large phase error of the order of 5°. However, due to the large linearity of the cores, the permeability is constant and so the phase error is constant in a wide range of measured currents. A constant phase error can easily be compensated up to a final accuracy of 0.05°.

However, the techniques mentioned here are not utilized for constructing larger CTs. The influence of the DC current component on the accuracy of larger CTs is usually not documented by manufacturers, and only a small number of papers have been published on this topic.

We have shown an effective way to measure DC currents in the power grid by protection of current transformers, which are already installed within the whole grid [15]. This is, in some cases, a preferable solution, when taking into account the costs and difficulties associated with installing new magnetic or optical DC current sensors at high-voltage lines and distribution stations [16,17,18]. Recently, we have also shown that by using a low-impedance excitation transformer and manual compensation, it is possible to keep 0.1% accuracy of the AC current measurement of these current transformers [19]. However, the aim to feedback-compensate the DC current component faces problems with stability. The main problems here are:

1. Large non-linearity and hysteresis of the CT when a DC current is present.
2. The measured and excitation current frequencies are in close proximity, so that it is difficult to separate them by an analog filter—the filter needs to be steep, which shifts the phase and compromises stability.

In this paper we show that automatic compensation of the DC flux is possible using a digital feedback loop. We also show that a DC-compensated CT can be made with single winding and without an excitation transformer. The design of our device is therefore simpler than the double-core solution described in [20,21].

The measurements in this paper are made on two current transformers:

1. A widely used measuring CT with a core made of oriented silicon steel—these measurements have already been described in [22].
2. CT with a nanocrystalline core with low remanence.

First, we verify the DC tolerance of both transformers, and how they work in fluxgate mode. On the basis of this benchmarking, we select one of them for the final device.

## 2. The Measured Transformers

The ordinary CT1 current transformer type CLA 2.2 (MT Brno, Czec Republic) has a 500 A/5 A current ratio and a rated output load of 5 VA, which corresponds to a nominal burden of 0.2 Ω. For this load, the error is below 0.1% from 5% to 120% of the nominal primary current (FS) of 500 A [15]. In our measurements, we load this CT by a 0.1 Ω sensing resistor to compensate for additional impedances in the measuring circuit. This CT represents the class of low-price, medium-performance devices. The main disadvantage is high remanence, which leads to a fatal error after the CT is magnetized by a DC current [3,23].

The second transformer, CT2, with a core made of nanocrystalline material, is representative of high-performance devices. The 140/100 × 20 mm core manufactured by NPAY has high permeability, low coercivity and low remanence. Due to this, it can easily recover from DC magnetization, even with a low AC measured current. This transformer has a 500 A/1 A current ratio and a rated output load of 1 VA, which corresponds to a nominal burden of 1 Ω. The lower output load of this transformer follows the tendency of devices for use as electronic power and energy meters.

## 3. DC Tolerance of Standard CT

In order to benchmark both measured transformers, we first measured the influence of the DC current on their accuracy. The accuracy was tested by comparing the CT to a Tettex 4764 current comparator (Tettex Instruments, Haefely Test AG, Basel, Switzerland), using the differential method. The error measurement was made by an SRS 830 digital lock-in amplifier (Stanford Research Systems, Inc., Sunnyvale, CA, USA). The accuracy of this method was verified by independent measurement using a Tettex 2767 automatic transformer (Tettex Instruments, Haefely Test AG, Basel, Switzerland) test set. The DC current was simulated by external 15-turn winding. In order to prevent loading of the transformer by the small impedance of the DC source, the DC source was AC-decoupled by a choke with large inductance.

**Figure 1:**
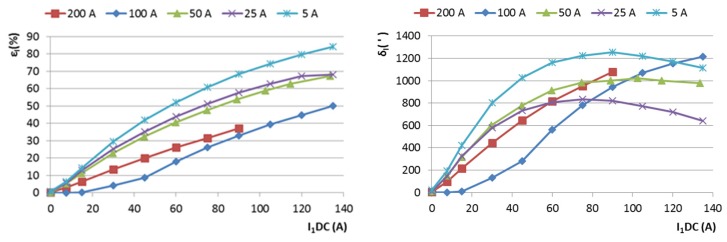
Ratio error ε_I_ and phase error δ_I_ of the 500 A/5 A current transformer CT1 as a function of the spurious DC current I_1DC_. AC measured current I_1AC_ is a parameter.

**Figure 2:**
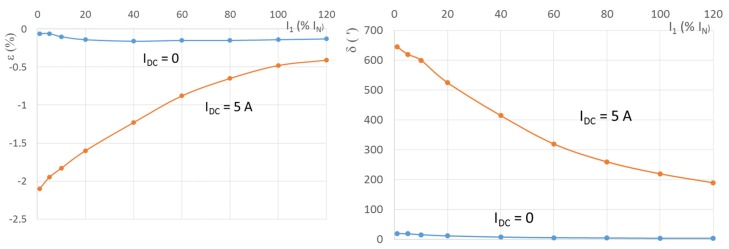
Ratio error ε_I_ and phase error δ_I_ of the 500 A/1 A current transformer CT2 for zero DC current and I_DC_ = 5 A as a function of measured current I_1_.

The results of this measurement are shown in Figure 1 for CT1, and in Figure 2 for CT2. The effect of a large DC current is devastating: a 50 A DC current in CT1 may cause 10% to 40% error in the current and power measurement. The amplitude error for a given I_DC_ decreases with increasing AC measured current. This is caused by a decrease in the relative asymmetry of the magnetization process. For AC around 100 A, the error reaches a minimum, and for larger AC currents it increases again. This is probably caused by the power limits of our amplifier.

The effect of the angular error on the error of the measured power depends on the power factor cos φ of the measured load. For a resistive load, the active power is equal to apparent power S, and the effect of δ is small. For small cos φ, the measured active power P = S cos(φ + δ) is very sensitive to phase error δ.

The error for CT2 is similar both in amplitude and in phase. In this aspect, the more expensive nanocrystalline material offers no advantage over a SiFe core.

## 4. Fluxgate Mode for the Detection of DC Current (Open-Loop Measurements)

**Figure 3:**
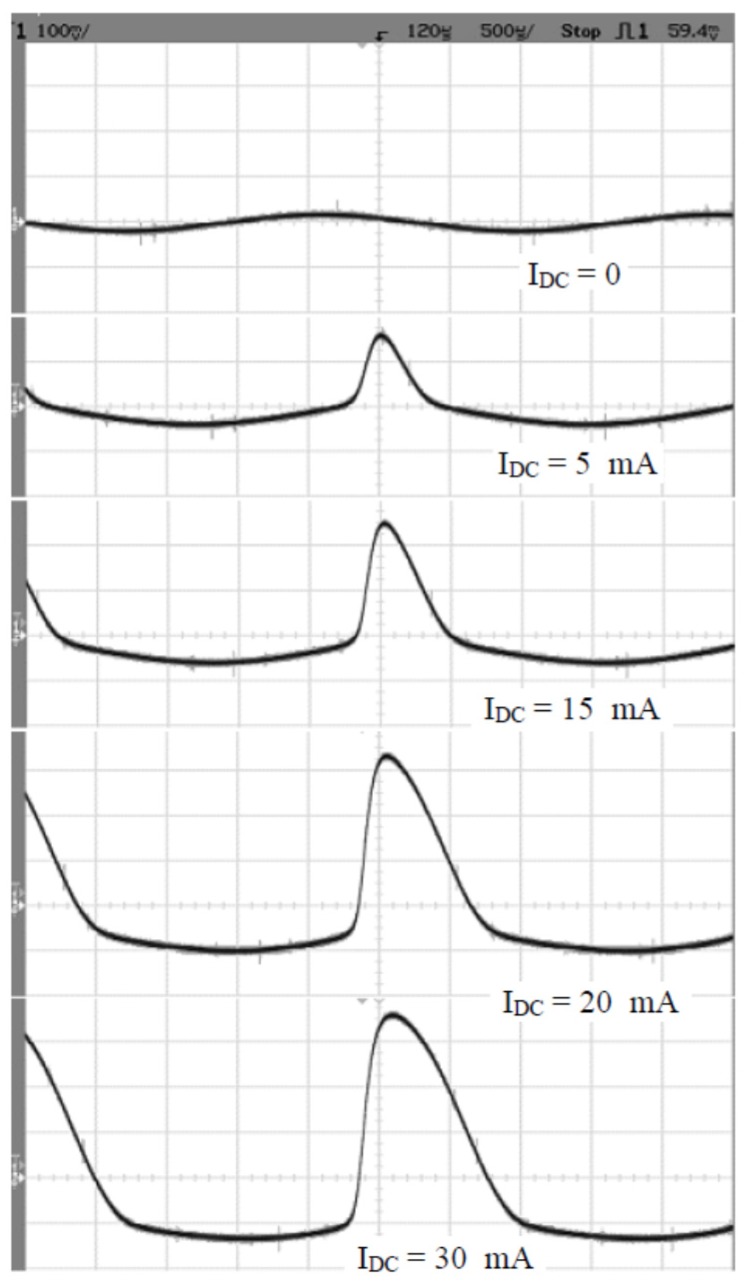
The I_exc_ for several values of I_DC_; the vertical scale is 1 A/div. Measured on CT2.

The DC flux component in the CT core can be detected by an additional sensor inserted into the core airgap, e.g., a Hall plate or a magnetostrictive element [24]. The basic disadvantage of the airgap is that it distorts the symmetry of the magnetic circuit, which results in dependence on the position of the current conductor in the sensor ring and increased leakage of the external magnetic fields into the sensing core, which cause sensitivity to external electric currents [25]. We therefore use a core without any airgap, and we detect the flux using the fluxgate effect [26,27]. The non-linear magnetization curve of the CT is shifted by the DC flux generated by the measured DC current, and it is no longer symmetrical. In our case, the CT is excited by f_exc_ = 370 Hz current I_exc_ injected into the secondary winding by a Kepco BOP 50–8 M power amplifier. The power amplifier has very small output impedance (R_out_ = 50 mΩ), which forces sinewave flux B. The non-symmetrical magnetization characteristic causes even harmonic components in the magnetic field intensity H inside the CT core. As H is proportional to I, we detect the second harmonic component of f_exc_ in the excitation current I_exc_ by the 1 SR 830 digital lock-in amplifier, which measures the voltage drop on the 0.1 Ω burden. The waveforms of I_exc_ for several values of I_DC_ are shown in Figure 3. Without the DC current component, I_exc_ is close to a sinewave, but the core is at the beginning of the saturation (top curve). Such a small excitation amplitude is unusual for a fluxgate, which usually requires deep saturation of the sensor core. In our case, low excitation current was used to save power. Even a small DC current component in the primary causes the core to be magnetized asymmetrically. As the excitation source has low impedance, the voltage is still forced to be a sinewave, and asymmetry appears in the secondary current. The measured parameter is the second harmonic current component. Larger values of the secondary DC current result in visible asymmetry caused by unipolar saturation. The current shape is similar to that of a short-circuited (current-output) fluxgate magnetic field sensor [28], but the theoretical description of the current sensor performance should be modified to fit the quantitative experimental results.

It should be noted that the power amplifier generates the excitation voltage, and it should simultaneously absorb 50 Hz secondary current without overload. In existing current comparators, these two functions are performed using separate excitation and sensing windings. In our device, we use only single winding, which brings many instrumentation difficulties, but allows us to use standard transformers, which are already installed within the grid.

**Figure 4:**
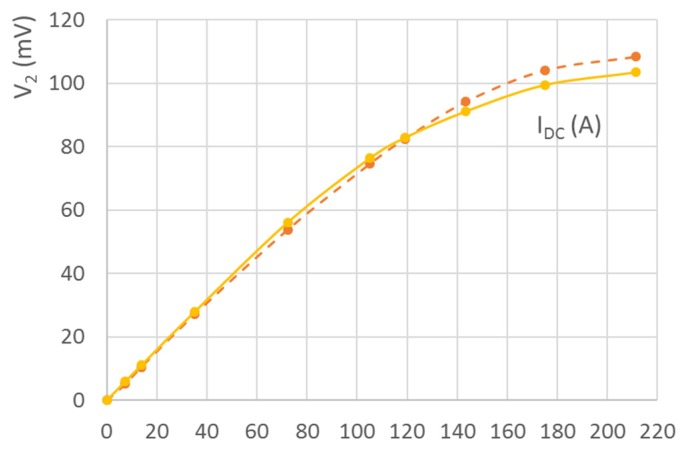
Second harmonic voltage V_2_ as a function of the DC current I_DC_ for R_1_ = 0 (dashed line) and R_1_ = 5 Ω (solid line).

Figure 4 shows the DC transfer characteristics measured on CT2: the dependence of the second harmonic component in the secondary current as a function of the DC component in the primary current measured in the open-loop. In the first experiments, we used separate excitation and sensing windings. We used sinewave current excitation and second harmonic detection in the induced voltage. With current excitation, we observed a large effect of the primary impedance on DC sensitivity. Here, we present results obtained for sinewave voltage excitation and second harmonic detection in the measured current. The graph confirms that stabilization of the sinewave excitation voltage is an effective strategy for reducing the dependence of the sensitivity on the grid impedance which, in real conditions, changes in time. R_1_ = 5 Ω is a realistic minimum grid impedance value.

The excitation current injected into the secondary winding is transformed into the primary circuit. This is unwanted, but the AC injected primary current I_inj_ drops rapidly with primary impedance: from 6.5 A for R_1_ = 0 to 100 mA for R_1_ = 5 Ω.

## 5. The Principle and the Design of the DC-Compensated CT

**Figure 5:**
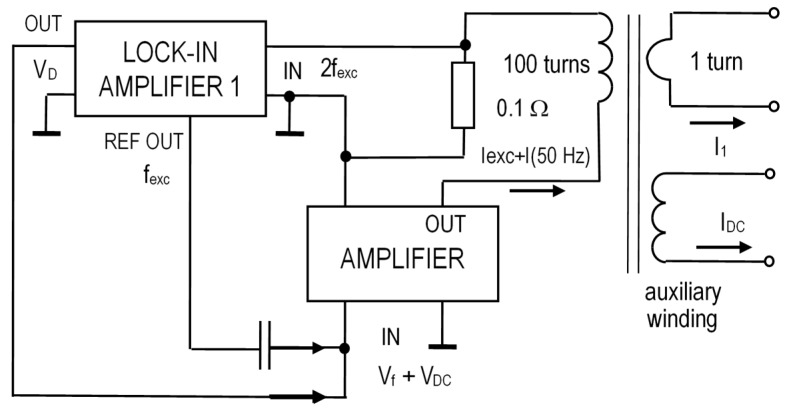
DC-compensated current transformer.

In this paper, we present a feedback-compensated DC/AC current transformer based on CT1 in order to demonstrate the potential of our solution to enhance the performance of a low-cost CT. The structure of our device is shown in Figure 5. The measured current I_1_ flows through a single-turn primary winding. This current has a DC component causing DC magnetic flux in the core. We measure this DC flux using the fluxgate principle by lock-in amplifier 1 as described in the previous section. The CT is excited by a Kepco BOP 50-8M power amplifier (KEPCO, INC. Flushing, NY, USA). This power amplifier is DC-coupled, and also serves for compensation of the DC flux component.

The analog output of lock-in amplifier 1 is fed back through the power amplifier to compensate the DC flux by the DC compensation current into the CT secondary. The digital nature of lock-in amplifier 1 allows the amplification, frequency response and phasing delay in the feedback loop to be set independently. This helps stable operation to be achieved even for highly non-linear characteristics.

## 6. The DC Current Response

**Figure 6:**
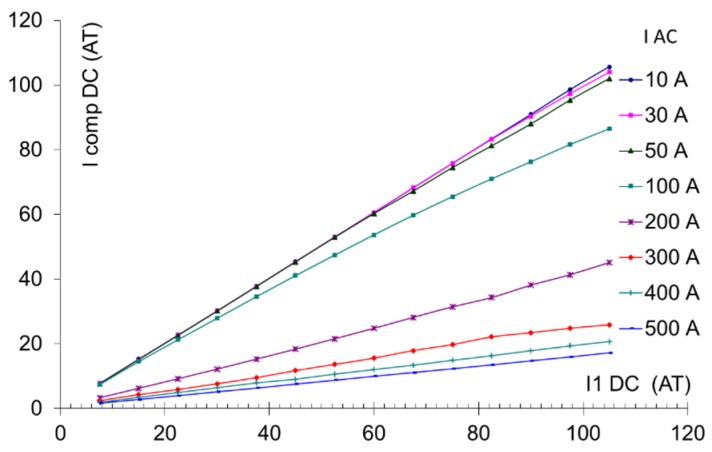
Closed-loop response to DC current I_1DC;_ AC measured current I_1AC_ is a parameter. Tested for low impedance in the primary circuit. The units for both axes are Ampere-Turns (magnetic voltage).

The feedback compensation current as a function of the measured DC current I_1DC_ is shown in Figure 6. The characteristic is quite linear, but the sensitivity drops significantly for measured AC currents larger than 100 A. We identified that this effect is strongly dependent on the impedance in the primary circuit. In the standard testing setup, the impedance at 370 Hz in the primary circuit of the tested CT is only 9 mΩ. This impedance was calculated as the ratio of the primary voltage of 0.408 V/370 Hz and the primary current of 43.9 A/370 Hz). In this case, the CT works practically in current transformer mode in the reverse direction, too, so that the excitation current injected into the secondary winding is transformed into a very large current (typically 50 to 100 A) in the primary circuit. The resulting excitation flux is very small, and the DC sensitivity depends strongly on the working point set by the measured AC current. Once the primary impedance is increased, the CT no longer works in current transformer mode for the excitation current, the excitation current injected into the primary circuit drops below 1 A, and the resulting excitation flux is much higher. The transformer is periodically saturated by the excitation current, even without any AC measured current. In this case, the CT works in the proper fluxgate mode. This makes the response to DC current more stable and less dependent on the value of the AC current.

Fortunately, the real grid has impedance between 0.2 and 40 Ω, which makes this problem less serious.

**Figure 7:**
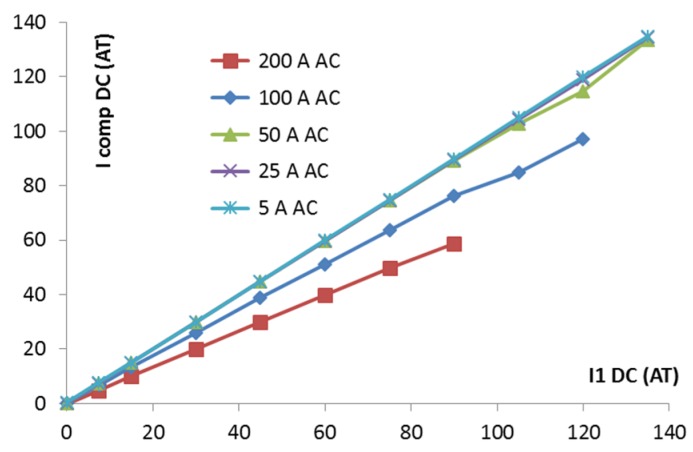
Closed-loop response to DC current for high impedance in the primary circuit. The parameter is the measured AC current I_1_.

The AC impedance of the power network had to be simulated for a 500 A AC current, which was above the limits of our experimental equipment. We improvised this by using a power inductor (a “choke”) formed by the secondary winding of the 60 kW transformer , manufactured by Agea Kull (Derendingen, Switzerland) (with the primary winding left open). The impedance of this inductor at an excitation frequency of 370 Hz was 0.08 Ω, sufficiently high to reduce the excitation leakage into the primary circuit to 5 A. The resulting characteristics are shown in Figure 7. At present, we are able to stabilize the feedback loop only for AC currents up to 200 A.

## 7. AC Precision

**Figure 8:**
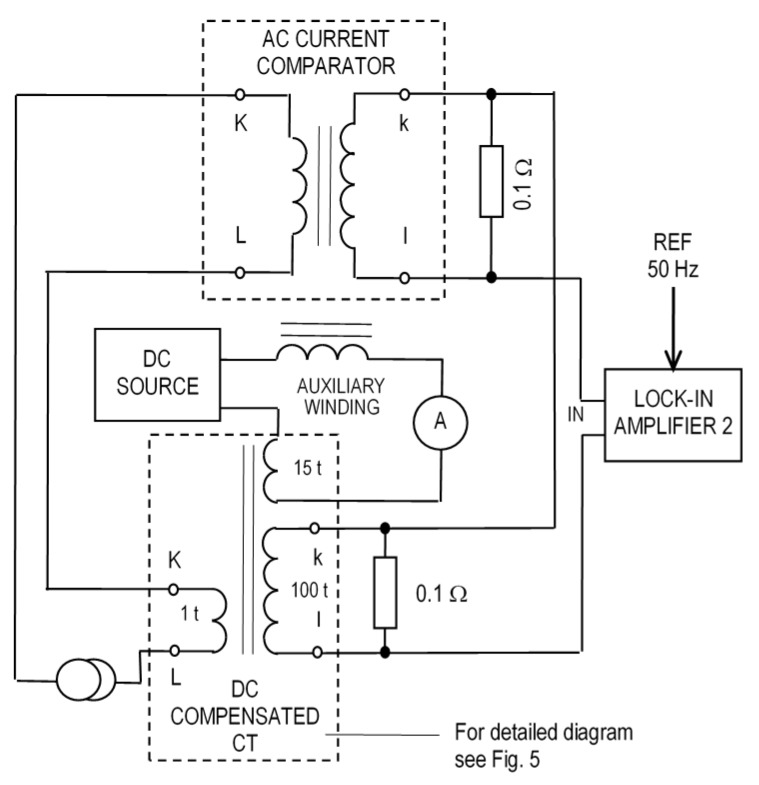
Measurement setup for testing the accuracy of the DC-compensated CT using a lock-in amplifier.

The DC-compensated CT was tested using the setup shown in Figure 8. For simplicity, our DC-compensated CT from Figure 5 is shown only as a simplified diagram. The effect of the DC current component is simulated by the auxiliary 15-turn winding supplied by the DC source. A serial inductor is used to prevent short-circuiting of the AC signal by this DC circuit. The measured AC current flows through the single-turn primary winding of this CT, and also through the current comparator. The current comparator is a standard of a current ratio with an accuracy of 10^−7^ [29]. While Lock-in Amplifier 1 is part of the CT electronics, Lock-in Amplifier 2 serves to measure the difference between the secondary currents of the CT under test and the current comparator. While the reference of Lock-in 1 is derived from the excitation signal, and the phase is adjusted for maximum sensitivity, the reference of Lock-in 2 is derived from the 50 Hz current source, and the reference phase is adjusted when measuring the full voltage across the burden of the comparator. Our setup subtracts two similar voltages across the burden resistors of the CT under test and the current comparator. Thus, the ADC of the digital lock-in amplifier measures only the current error, not the current value. This gives much better accuracy than when both currents are measured separately and only the digital value is subtracted, as was implemented in [30]. A similar setup with a single burden resistor can also be used.

**Figure 9:**
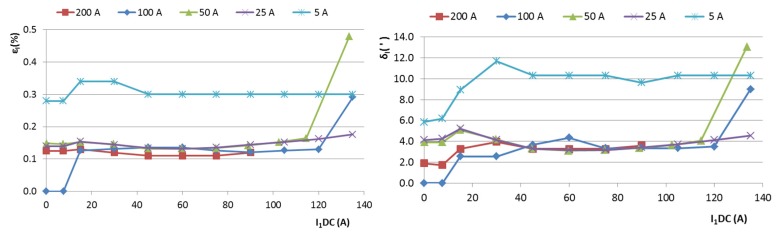
Amplitude and phase errors of the DC-compensated current transformer as a function of the primary DC current.

The measured errors are shown in Figure 9. For extreme values of DC currents combined with high AC currents, the transformer worked in nonlinear mode and the feedback loop was on the border of stability. This is indicated by the steeply increasing error, as shown on the curves for 50 A and 100 A AC. For 200 A AC, the feedback loop could be stabilized only for DC currents below 90 A.

## 8. Conclusions

In this paper, we have shown for the first time that automatic compensation of the DC current in the current transformer is possible using a digital feedback loop. We were able to use the same DC-coupled power amplifier for feedback compensation and for excitation. A DC-compensated CT can be made with single winding and without an excitation transformer. The principle also works for a high-end CT with a nanocrystalline core. However, we have demonstrated that a low-cost CT with an FeSi core can also be used successfully for this device. In the extreme case, we reduced the CT amplitude error by DC compensation from 60% to 0.15%. Even in the presence of full-scale AC measured current, the amplitude of the spurious DC current can be indicated with sufficient accuracy, without the need to install new sensors.

The proposed method can increase the accuracy of the energy meters and can simultaneously monitor the DC current for protection purposes. We verified the feasibility of our method by laboratory tests using expensive and large instruments, such as a lock-in amplifier and a power amplifier. For industrial applications, these instruments should be replaced by custom-made circuits to reduce the cost, size and power consumption.
